# Turnover Intention and Its Relationship With Work‐Related Factors Among Nurses in Spain: A Cross‐Sectional Study

**DOI:** 10.1155/jonm/7697811

**Published:** 2026-05-09

**Authors:** Lucia Rocio Camacho-Montaño, Sonia Betsabé Gómez-Arribas, Maria Teresa Moreno-Casbas, Andrea Ropero-Sánchez, Leticia Bernués-Caudillo, Paloma Calleja-Toledano, Alda Recas-Martín, Javier Zamora, Javier Padilla-Bernáldez

**Affiliations:** ^1^ Nursing and Health Research Unit (Investén-isciii), Instituto de Salud Carlos III, Madrid, Spain, isciii.es; ^2^ Gabinete de la Secretaría de Estado de Sanidad, Ministerio de Sanidad, Madrid, Spain; ^3^ CIBER of Frailty and Healthy Ageing (CIBERFES), Instituto de Salud Carlos III, Madrid, Spain, isciii.es; ^4^ University of Vic – Central University of Catalonia (UVic-UCC), Catalonia, Spain; ^5^ Spanish Centre for Evidence-Based Nursing and Healthcare: A Joanna Briggs Institute Centre of Excellence, Instituto de Salud Carlos III, Madrid, Spain, isciii.es; ^6^ Clinical Biostatistics Unit, Hospital Ramón y Cajal, IRYCIS, Madrid, Spain, irycis.org; ^7^ CIBER de Epidemiología y Salud Pública (CIBERESP), Madrid, Spain, ciberesp.es; ^8^ Secretario de Estado de Sanidad, Ministerio de Sanidad, Madrid, Spain

**Keywords:** cross-sectional study, nurses, personnel turnover, push–pull factors, turnover intention, working conditions

## Abstract

**Background:**

Nurse turnover intention is a multifactorial construct shaped by individual, occupational, organizational, and policy‐level factors. Although widely studied in some regions, evidence from Southern Europe, particularly Spain, remains scarce. Understanding these factors in diverse healthcare contexts is essential for designing effective retention strategies that are both locally relevant and internationally informative.

**Objective:**

This study aims to explore turnover intention prevalence and associated factors among nurses across different care settings in Spain.

**Design:**

A cross‐sectional study.

**Settings:**

Primary care, hospitals, emergency services, or social healthcare settings.

**Participants:**

A total of 20,316 actively employed nurses.

**Methods:**

An online survey was disseminated by the Spanish Ministry of Health and other institutional channels. Turnover intention was the primary outcome and was measured with a single item asking whether nurses intended to leave the profession within the next 10 years (yes/no). Work‐related variables and perceptions of care quality and patient safety were also collected. Multivariable logistic regression was used to identify key predictors across care settings.

**Results:**

Of the 20,316 participants, most were women (84.8%) and 50.3% were under 35 years. Overall, 39.58% reported an intention to leave the nursing profession within 10 years. Turnover intention was significantly associated with perceptions of care quality (OR = 1.706, *p* < 0.001) and patient safety (OR = 1.809, *p* < 0.001), regional disparities, and temporary employment contracts (OR = 1.333, *p* < 0.001). In primary care, working as a generalist and on afternoon shifts increased turnover risk, while in hospitals, long shifts (> 7.5 h) were influential.

**Conclusions:**

This study provides novel insights into nurse turnover intention in Spain, highlighting the interplay of modifiable institutional factors and regional disparities in a decentralized health system. Turnover intention was strongly associated with organizational conditions, reinforcing the urgent need for tailored, context‐sensitive retention strategies. Standardizing definitions, measurements, and temporal frameworks remains critical for advancing comparative research and developing effective, evidence‐based interventions to strengthen the nursing workforce.

**Summary**
 What is already known
• Nurse turnover intention represents a critical warning signal and an intervenable window between intention and actual turnover behavior.
• Rates of turnover intention vary widely across countries, influenced by working conditions, contracts, education, and salaries.
• Turnover intention is a complex construct shaped by individual, organizational, and policy‐level determinants.
 What this paper adds
• This is the largest study to date on turnover intention in Spain, covering multiple care settings. Nearly 40% of Spanish nurses reported a turnover intention within 10 years, with substantial disparities reflecting the decentralized health system.
• Nurses’ perceptions of care quality, patient safety, and employment instability were identified as key modifiable factors influencing turnover intention.
• The study highlights heterogeneity in definitions and measurements of turnover intention, emphasizing the need for standardization to develop effective evidence‐based retention strategies.

## 1. Background

Projections estimate a global shortfall of 7.6 million nurses by the year 2030, highlighting a critical challenge for health systems [1]. The COVID‐19 pandemic further intensified this crisis by causing an abrupt and considerable increase in the demand for nursing professionals [2]. Turnover refers to the actual departure from a job, whether voluntary or involuntary, including retirement, contract termination, or medical leave [3–5]. Actual turnover exacerbates the existing shortage of nurses, a problem that is already critical worldwide [3, 5, 6] and further delays its resolution [7]. The term turnover intention (TI) is defined as the conscious reflection and planning process regarding the decision to exit one’s current role, employing institution, or the nursing profession entirely [3–5]. This intention constitutes a critical cognitive process that precedes the actual turnover, representing a key warning signal, and an intervenable window between intention and behavior. It is therefore considered a robust indicator for predicting turnover behavior [8–10].

Evidence highlights a sustained upward trend in nursing TIs. At the global level, estimates indicate wide variability in TI rates among nurses, ranging from 4% to 54% [3, 11], with an overall turnover rate of 18.7% recorded in 2022 [12]. In Europe, the Registered Nurse Forecasting (RN4CAST) project revealed that between 20% and 50% of nurses reported an intention to leave their current position [13], while another study encompassing 10 European countries found that 33% of nurses reported considering leaving the profession [9]. This persistent trend contributes to a widening gap between the growing demand for healthcare services and the constrained supply of qualified nursing personnel [9, 14].

Turnover within healthcare organizations entails substantial economic costs, estimated to be up to three times the average nurse salary per each departure [6]. Additionally, high turnover diminishes productivity and performance among remaining staff, as reduced nurse availability leads to increased workload and extended shifts, which in turn elevate the risk of further resignations [6, 9]. This cyclical shortage–turnover phenomenon exacerbates workforce instability [6]. Elevated turnover rates are also correlated with increased fatigue, burnout, stress, decreased job satisfaction [6, 12], and a negative work environment [4]. Collectively, these factors adversely impact patient care, compromising safety and quality of health services and contributing to increased mortality rates [7, 12, 13].

TI is a multifactorial construct shaped by the interplay of individual, occupational, organizational, and policy‐level determinants [3, 6, 15]. Theoretical models [3, 16–19], including the job demands–resources (JD‐R) framework adapted to the European Union context [16], highlight burnout and job satisfaction as key predictors of TI, both influenced by a constellation of interrelated factors [11, 17, 18]. Additionally, individual motivation has been identified as a critical determinant, alongside social dynamics and characteristics of the work environment [3]. Several European studies have examined determinants of TI [17, 19], emphasizing the pivotal role of work‐related factors in nurse retention [6]. Among these, perceived staffing shortages, resource constraints, limited time for direct patient care, missed nursing care, and negative perceptions of care quality stand out. Furthermore, factors such as high workload, role ambiguity, rotating or night shifts, extended overtime, and insufficient supervisory support have been consistently associated with increased TI [3, 7, 19, 20].

Although global nurse turnover rates have been estimated, considerable variability exists between countries [2, 7, 14]. Europe currently faces a critical shortage of healthcare professionals, an aging workforce, and growing challenges in professional training [18]. Identification of TI and its determinants is essential for designing targeted interventions to strengthen workforce retention [3, 6, 13, 21, 22]. In response, the World Health Organization (WHO) has called for urgent implementation of evidence‐based policies to identify best practices in retention [23]. However, research on the European workforce, and specifically on Spanish nursing, remains limited [17, 18].

The decentralized structure of the Spanish National Health System (SNS) provides a strong rationale for this study. TI is highly context‐sensitive [11], and Spain’s decentralized SNS may generate substantial regional disparities, yet the extent to which these disparities influence nurses’ intention to leave remains unclear. To date, no large‐scale study has systematically examined TI among Spanish nurses across autonomous communities (ACs). This underscores the relevance and urgency of conducting a region‐specific study to capture workforce heterogeneity and to inform context‐sensitive retention strategies. To bridge this gap, this study identifies the specific factors driving TI among Spanish nurses. In this context, it forms part of a strategy led by the Healthcare Committee within the Ministry of Health’s Health Division aimed at supporting workforce planning for the SNS by providing a comprehensive assessment of TI and its associated factors and contributing to the development of effective staff retention strategies within the SNS [24].

## 2. Aims

The aim of this study is to comprehensively explore the prevalence of TI among nurses in Spain and to analyze the multifactorial determinants that underlie this phenomenon.

1. What is the prevalence of TI among Spanish nurses?
2. Are sociodemographic characteristics, work‐related factors, and perceptions of care quality and patient safety associated with higher TI?
3. Which group of factors (sociodemographic, work‐related, or care quality–related) explains the greatest proportion of variance in TI?

## 3. Methods

The study employed a cross‐sectional design following the STROBE Statement. For additional details, see the Supporting file (STROBE Statement—Checklist of items that should be included in reports of cross‐sectional studies) (available here). It constitutes a secondary analysis of data originally collected within a nationwide macro‐survey commissioned by the Ministry of Health and administered to practicing nurses in Spain as part of the Strategic Framework for Nursing Care [24]. While the original project aimed to provide a comprehensive profile of the nursing workforce, the present analysis specifically examines the determinants of TI among nurses in Spain.

### 3.1. Sample Size

Based on the formula:

(1) N=μα2·π·1−πδ2,

with *π* = 0.48, the estimated proportion of TI [10], *μ*_*α*_ = *Z* score for 99% confidence, and *δ* = 0.03 (margin of error), the minimum required sample size was 1846. Considering a 20% dropout rate, a sample size was adjusted to 2308 participants.

### 3.2. Data Collection

The survey was developed under the auspices of the Healthcare Committee within the Ministry of Health’s Health Division, aimed at supporting workforce planning for the Spanish SNS. The instrument was collaboratively designed by an expert panel composed of academic researchers and clinical nursing experts. The questionnaire items were selected to study the factors reported by nurses in Spain that are recognized in the literature as critical for workforce planning and management. Some of the items were adapted from another project conducted as part of the Report on the Supply and Needs of Medical Specialists [25]. The panel initially assessed the relevance, clarity, and comprehensiveness of the items in relation to the study objectives. Their feedback was used to refine the questionnaire and ensure its content validity. A pilot study was conducted with a demographically homogeneous cohort of nurses to assess feasibility, estimating an average completion time of 30 min.

The self‐administered questionnaire consisted of 105 items structured into seven thematic domains: (1) sociodemographic and professional profile; (2) educational and continuous professional development; (3) TI; (4) occupational characteristics; (5) last shift–related variables; (6) patient care data during the last shift; and (7) perceptions of patient safety and care quality.

Data were collected via an online survey platform REDCap (Research Electronic Data Capture) between May 7 and May 31, 2024. Recruitment leveraged multiple institutional channels, including the Spanish general council of nursing, the Ministry of Health, the Instituto de Salud Carlos III (ISCIII) and official digital communication networks, ensuring broad representation.

Eligibility criteria encompassed nurses who (1) were aged between 18 and 60 years; (2) possessed a bachelor’s degree or diploma in nursing; (3) held active employment as generalist nurses, specialist nurses, or resident specialist nurse (EIR, using the Spanish‐language acronym); (4) were currently practicing in primary care (PC), hospitals, emergency services, or social healthcare settings; and (5) provided written informed consent. Participants with incomplete responses to any survey item were excluded to maintain a dataset free of missing data (NAs). Furthermore, nurses aged over 55 who indicated an intention to leave the profession within the next 5–10 years were excluded due to their proximity to retirement age; their reported intention likely reflected retirement rather than voluntary turnover. This exclusion was applied to ensure that the analysis was focused on voluntary TIs and was not confounded by retirement‐related departures.

### 3.3. Outcomes

The primary outcome of this study was TI, assessed using the following item: “Do you usually think about leaving the nursing profession in the coming years (including if you have not thought about it but believe you would leave if you had another job opportunity)?” Multiresponse options were: “Yes, within 2 years”; “Yes, within 3 years”; “Yes, within 5 years”; “Yes, within 10 years”; and “No, I do not currently plan to leave the profession.” For analytical purposes, the variable was dichotomized as Yes/No (Yes = 1/No = 0), with any “Yes” response within the next 10 years classified as “Yes.”

• Sociodemographic variables: age, gender, and educational level. Age was categorized into three groups: under 35 years, 35–55 years, and over 55 years.
• Work‐related variables: professional level, type of employment contract, shift worked (day, evening, and night), hours worked, and number of patients as an indicator of workload. Hours worked were categorized based on the median value. Number of patients was categorized according to quartiles (1st quartile, median, and 3rd quartile).
• Nurses’ perception of work‐related variables: perception of adequate staffing (Yes/No), perception of sufficient number of nurses (Yes/No), occurrence of missed nursing care activities due to lack of time (Yes/No), frequency of patient safety incidents occurring “once or more times per week” (Yes/No), perceived quality of nursing care provided (very good/good/fair/poor/very poor), and perception of patient safety and quality of care during the last shift worked (very good–good/fair/poor/very poor). Both variables were regrouped into three categories (good/fair/poor).

### 3.4. Data Analysis

Preprocessing of the full project dataset was conducted to apply the study’s inclusion criteria. Cases with missing data were excluded, and all independent variables were converted into categorical factors. Three continuous variables were categorized for analysis: age, hours worked, and number of patients. Age was grouped into three categories, young adults, adults, and those approaching retirement, to approximate career stages; hours worked and number of patients were categorized based on distributional considerations. Categorical variables are reported as frequencies and percentages. Univariate analyses were performed using appropriate statistical tests to compare differences in proportions between nurses reporting TI and those who did not. For statistically significant associations, post hoc analyses were carried out using standardized residuals and Holm’s sequential Bonferroni method to adjust for multiple comparisons. The adjustments were applied separately for each variable. The family of tests corresponded to all possible pairwise comparisons between categories, reaching a maximum of 136 tests for the analysis of ACs (17 categories). Subsequently, a logistic regression model was constructed with TI as the dependent variable (coded as 1 = intends to leave; 0 = does not intend to leave).

Factors previously associated with TI in the literature [3, 8, 19, 26] and identified in univariate analyses were included in the model, encompassing 17 work‐related variables. Prior to analysis, the variance inflation factor (VIF) was calculated to assess potential multicollinearity among the independent variables. To determine the optimal model, alternative models were evaluated based on likelihood ratio tests, adjusted *R* ^2^, Akaike information criterion (AIC) values, and model goodness of fit assessed by the Hosmer–Lemeshow test. Backward stepwise selection was employed to identify the final model, confirming that the full model provided the best overall fit. The effect of each independent variable in the final multivariable analysis was expressed as odds ratios (ORs) with corresponding 95% confidence intervals (CIs). In addition to the full model, a block‐wise analysis was conducted to evaluate the relative contribution of each block: (1) sociodemographic block: gender, age, education level, and region; (2) work‐related block: type of institution, current position, number of patients, contract type, shift, hours worked, and care setting; and (3) care quality and safety block: perception of adequate staffing, missed nursing care, quality of care, patient safety, and occurrence of incidents in the unit. Model comparison based on AIC and pseudo‐*R* ^2^ values demonstrated progressive improvement with the addition of explanatory blocks. Furthermore, a binary logistic regression model stratified by institution type PC or hospitals was conducted. A *p* value < 0.05 was considered statistically significant. All analyses were performed using RStudio Version 4.4.0.

### 3.5. Ethical Issues

This population‐based survey was executed ensuring strict adherence to respondent anonymity and confidentiality, in full compliance with current personal data protection laws, including the General Data Protection Regulation and the ethical principles outlined in the Declaration of Helsinki. Informed written consent was obtained from all participants prior to data collection. The study protocol was reviewed and approved by the Ministry of Health.

## 4. Results

**FIGURE 1:**
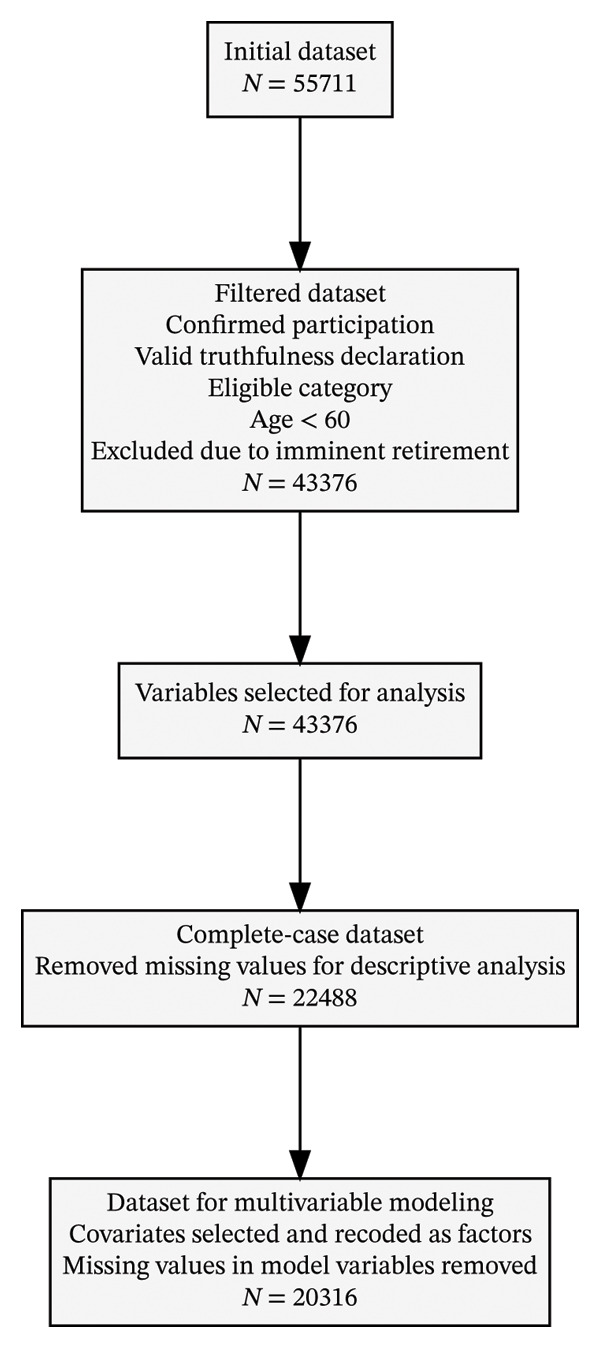
Flow diagram.

**FIGURE 2:**
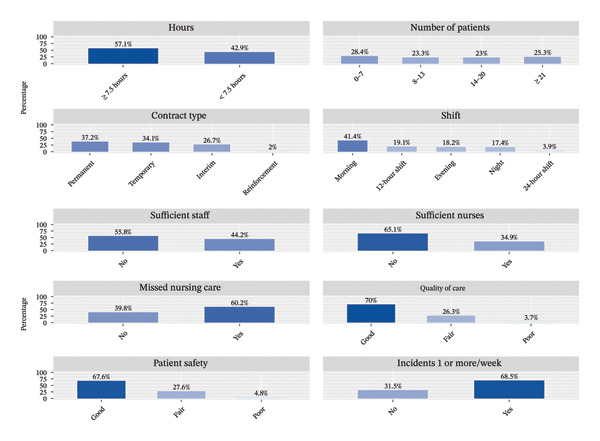
Nurses’ working environment characteristics and their perceptions of patient safety and quality of care during the last shift.

From the original dataset, a subsample of 20,316 nurses was selected (Figure 1). The cohort was predominantly female (84.81%) and relatively young (50.26% under the age of 35), and nearly one‐third (29.44%) possessed a master’s degree, while 21.71% were EIR nurses. Most participants worked in hospital settings (65.51%). The overall prevalence of TI from the nursing profession over the next 10 years was 39.58%, including 17% intending to leave within 2 years and 4% within 3 years (Table 1; Supporting file 1: Figure 2). Detailed descriptive analyses of demographic and professional characteristics are presented in Table 1, and perceptions of patient safety and quality of care are summarized in Figure 2.

**TABLE 1:** Sociodemographic and occupational characteristics of the study participants (*n* = 20,316).

Variable	Level	Total
Gender	Female	17,229 (84.81%)
	Male	3087 (15.19%)

Age	0–34 years	10,210 (50.26%)
	35–54 years	9234 (45.45%)
	55–60 years	872 (4.29%)

Educational level	Bachelor’s degree	9724 (47.86%)
	Master’s degree	5982 (29.44%)
	EIR	4410 (21.71%)
	Doctorate	200 (0.98%)

Care setting	Hospital	13,309 (65.51%)
	Primary care	4753 (23.4%)
	Emergency services	1650 (8.12%)
	Social healthcare	604 (2.97%)

Current position	Generalist	17,572 (86.49%)
	Specialist	2744 (13.51%)

Area of practice	Urban	16,698 (82.19%)
	Both	2147 (10.57%)
	Rural	1471 (7.24%)

Turnover intention in the next…	2 years	3451 (16.99%)
	3 years	743 (3.66%)
	5 years	1883 (9.27%)
	10 years	1965 (9.67%)

*Note:* EIR (using the Spanish‐language acronym): resident specialist nurse.

Most of the nurses were employed in the regions of Madrid, Andalusia, and Catalonia. Percentages by AC can be found in Supporting file 1: Figure 1. Detailed post hoc univariate analyses comparing proportions by TI are provided in Supporting file 2: Table 1.

A binary logistic regression model was constructed to examine factors associated with TI (0 = No, 1 = Yes). No multicollinearity was detected (all VIFs ≤ 2). The model showed adequate fit (Hosmer–Lemeshow test *χ* ^2^ = 14.99, *p* = 0.308, AIC = 25382.15), with overall performance supported by a likelihood‐ratio *χ* ^2^ (G^2^) = 1989.75, a Nagelkerke *R* ^2^ = 0.126, and an adjusted *R* ^2^ = 0.093.

Geographical location was one of the strongest contributors to TI, and the highest regional odds (OR > 2) were observed in Madrid, Canary Islands, Galicia, and Balearic Islands relative to Navarre. Perceptions of patient safety and care quality also emerged as the significant drivers: poor perception of patient safety (OR = 1.809), poor care quality (OR = 1.706), frequent safety incidents (OR = 1.329), missed nursing care (OR = 1.331), and perceived staffing inadequacy (OR = 1.193) significantly increased the odds of intending to leave (all *p* < 0.001). Work‐related factors such as temporary contracts (OR = 1.333), shifts exceeding 7.5 h (OR = 1.321), and evening shifts (OR = 1.253) were also significant (*p* < 0.001). Holding a master’s degree (OR = 1.395), being under 35 years of age (OR = 1.211), and male gender (OR = 1.145) were significantly associated (*p* < 0.001) as detailed in Table 2.

**TABLE 2:** Logistic regression analysis of factors associated with nurses’ turnover intention.

Variable (ref)		OR	Std. error	*z*	*p*	95% CI	
						Lower	High
(Intercept)		0.087	0.135	−18.165	0.000	0.067	0.113

Male (ref: female)		1.145	0.042	3.219	0.001	1.054	1.244

0–34 years (ref: 35–54 years)		1.211	0.037	5.236	0.000	1.127	1.302

Master (ref: no)		1.395	0.032	10.366	0.000	1.310	1.486

Institution (ref: hospital)	Social healthcare	0.779	0.096	−2.604	0.009	0.645	0.939
	Primary care	1.046	0.050	0.906	0.365	0.948	1.155
	Emergency services	0.853	0.061	−2.624	0.009	0.757	0.960

Generalist (ref: specialist)		1.117	0.052	2.125	0.034	1.009	1.238

Contract type (ref: permanent)	Interim	1.235	0.042	5.032	0.000	1.138	1.342
	Temporary	1.333	0.045	6.449	0.000	1.221	1.455
	Reinforcement	1.169	0.112	1.396	0.163	0.938	1.455

Shift (ref: morning)	Evening	1.253	0.044	5.177	0.000	1.151	1.365

≥ 7.5 h (ref: < 7.5)		1.321	0.044	6.323	0.000	1.212	1.440

Number of patients (ref: 0–7)	8–13	0.884	0.044	−2.826	0.005	0.811	0.963
	14–20	0.952	0.046	−1.051	0.293	0.869	1.043
	≥ 21	1.036	0.050	0.704	0.481	0.939	1.142

Sufficient staffing (ref: yes)		1.193	0.036	4.886	0.000	1.111	1.281

Missed nursing care (ref: no)		1.331	0.036	7.914	0.000	1.240	1.429

Quality of care (ref: good)	Fair	1.441	0.042	8.781	0.000	1.328	1.563
	Poor	1.706	0.099	5.383	0.000	1.405	2.073

Patient safety (ref: good)	Fair	1.284	0.041	6.130	0.000	1.186	1.391
	Poor	1.809	0.089	6.656	0.000	1.520	2.156

Incidents ≥ 1/week (ref: no)		1.337	0.035	8.318	0.000	1.249	1.432

*Note:* Odds ratios (ORs), standard errors (Std. error), *z*‐values (*z*), *p* values (*p*), and confidence intervals (CIs) are reported from the logistic regression model assessing factors associated with nurses’ turnover intention from the profession. Ref: reference category. Statistically significant associations are considered at *p* < 0.05. Variables with OR > 1 indicate increased odds of turnover intention, whereas OR < 1 indicates decreased odds and an OR = 1 indicates no effect.

### 4.1. Block‐Wise Logistic Regression Analysis

The model including only care quality and safety perceptions showed moderate performance (AIC = 26,020; *R* ^2^ = 0.08). Adding work‐related variables to care perceptions improved model fit (AIC = 25,796; *R* ^2^ = 0.10), while inclusion of sociodemographic factors further increased explanatory power (AIC = 25,499; *R* ^2^ = 0.12). The full model achieved the best fit (AIC = 25,401; *R* ^2^ = 0.13). Nevertheless, Nagelkerke *R* ^2^ values ranging from 0.08 to 0.13 indicate modest overall explanatory power suggesting that TI is influenced by additional factors not captured in this model. See Table 3 for details.

**TABLE 3:** Comparison of explanatory models for turnover intention by sociodemographic, work‐related, and care quality domains.

Model	AIC	*R* ^2^ Nagelkerke	Adjusted *R* ^2^
Base	27,278		
Sociodemographic	26,542	0.051	0.038
Work‐related	26,777	0.035	0.026
Care quality	26,020	0.082	0.061
Sociodemographic + work‐related	26,272	0.071	0.052
Work‐related + care quality	25,769	0.099	0.074
Sociodemographic + care quality	25,499	0.117	0.087
Full model (all blocks combined)	25,382	0.126	0.093

Overall, the strongest predictors of TI were geographical location (several ACs with OR > 2), and poor perceptions of patient safety and quality of care, temporary contracts, longer working hours, and evening shifts showed the largest effect sizes. Holding a master’s degree and younger age were also associated with higher odds of TI, although their impact was smaller in magnitude compared with care‐ and work‐related factors. For further details, consult Supporting file 3: Table 1.

### 4.2. Stratified Logistic Regression Model

A binary logistic regression model was stratified by type of healthcare institution (hospitals or PC). This analysis revealed that the core drivers of TI, regional disparities and poor perceptions of care quality and patient safety, remained consistent. In PC settings, generalist roles (OR = 1.577), afternoon shifts (OR = 1.473), and having a temporary contract (OR = 1.426) were particularly strong predictors of TI (*p* < 0.001). In contrast, hospital settings were extreme regional variations, notably in Melilla (OR = 3.168), shifts exceeding 7.5 h (OR = 1.371). See Supporting file 3: Figure 1.

**FIGURE 3:**
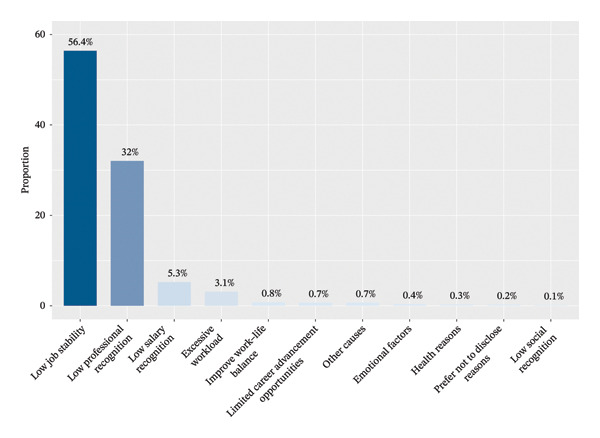
Reasons for turnover intention.

Nurses who reported a TI were asked about the underlying reasons for this decision. The most frequently cited factors were predominantly professional in nature, including lack of job stability (56.54%), insufficient professional recognition (31.54%), and inadequate salary (5.65%), as well as an excessive workload (3.55%). Figure 3 provides details.

## 5. Discussion

The aim of this study was to determine the prevalence of TI from the nursing profession among nurses in Spain and to identify the factors associated with this phenomenon. To date, this is the first large‐scale study conducted in Spain to report such a high level of TI from the nursing profession.

In our study, 39.6% of nurses reported an intention to leave the nursing profession within the next 10 years, a rate consistent with other recent estimates [16, 27], but representing a substantial increase compared with the 5% reported in 2013 across European countries [28]. While TI varies considerably across geographical and professional contexts [18], Spain appears among the European countries with the highest risk [29]. Although international comparisons are constrained by heterogeneous definitions and time frames (detailed in Supporting file 4: Tables 1 and 2), European studies report lower rates than those observed in our analysis, ranging from 11.7% for the following year [10] to 17.7% within the next 6 months [11], indicating that shorter time frames are associated with higher reported intentions to leave [10, 11, 17, 18]. In Spain, short‐term TI (≤ 12 months) remains unknown.

Beyond these rates, TI in Spain is associated with a complex interplay of multilevel determinants. At the system level, AC of employment emerged as a key factor in our model, underscoring the importance of contextual and organizational conditions. This finding can be partly explained by the decentralized structure of the Spanish healthcare system [30]. The devolution of management to 17 ACs has led to substantial regional disparities in nursing staffing levels, working and salary conditions, residency training positions, and specialty recognition, as documented in a report from Ministry of Health reports [31]. Another relevant factor may be the mismatch between the high educational attainment of Spanish nurses (EIR training and master’s degrees) and actual professional rewards. In Spain, specialist nurses are often employed in generalist roles, which make them more susceptible to TI compared with nurses in other European countries [31]. This aligns with Enea et al. [17], who explained cross‐national differences in TI to broader structural factors such as working conditions, cost‐adjusted salaries, employment rates and labor market, and educational system characteristics.

According to previous studies, countries where nurses report better working conditions tend to have lower TI rates [32], highlighting the central role of organizational, work environment, and resource‐related factors in nurses’ decisions to leave the profession and nurse retention [6, 19]. At the organizational level, our study found that worse working conditions, such as temporary or interim contracts and evening shifts, were associated with a higher likelihood of intending to leave the profession. However, the effect of night shifts was not significant in the multivariate analysis, despite being significant in univariate tests; a similar pattern was observed for relief contracts. Consistent with prior literature, both evening and night shifts have been linked to increased TI [3, 7, 28], specifically, night shifts to increased TI, whether as a general risk factor [17] or when exceeding a frequency of two shifts per week [8, 33]. Similarly, Ivziku et al. [11] reported lower TI among nurses working day shifts, whereas longer working hours were associated with higher TI, in line with our findings.

Our study revealed a potential protective effect when nurses cared for fewer than 14 patients, an effect that disappeared once this threshold was reached. This implies that high patient loads alone may not drive TI and could instead relate to care complexity [34]. In hospital settings, Enea et al. [17] identified emergency departments as a risk factor; however, Mafula et al. [7], in a meta‐analysis, found that TI was lower among those in hospital emergency units. Similarly, our findings diverge, showing a potential protective effect for nurses in emergency services, compared to other hospital nurses, against TI. The difference in our results may be due to the organizational structure of the SNS, in which emergency services are not confined to hospital settings but are integrated across all levels of care, including PC through dedicated out‐of‐hospital emergency units [35]. High turnover rates have been a critical issue in nursing homes, with turnover reported to be higher compared to other settings [6]. In contrast, our findings suggest a potential protective effect for nurses working in social healthcare settings. This deviation from previous literature may be explained by the composition of our sample, in which 66% of participants belonged to hospitals, which was the subgroup with the highest reported TI. Rahnfeld et al. [35, 36] further proposed that, among geriatric nurses, factors such as greater social interaction and opportunities for collective breaks may act as buffers, mitigating the impact of high job demands, including social conflicts and time pressure.

Our stratified models by institution type further illustrate how the effects of various factors differ depending on the clinical setting. The effect of age was particularly pronounced in PC compared to hospitals. In the literature, retaining young nurses, especially in hospitals [6], is a key challenge. Specific work‐related factors also played a role: in PC, working as a generalist increased TI by 56% compared to specialists, an effect that was not statistically significant in hospitals. The evening shift was associated with higher odds of TI in PC (+48%) compared to the day shift, while in hospitals, the increase was +21%. Additionally, the 12‐hour shift in hospitals was a protective factor, reaching statistical significance (OR = 0.836, *p* < 0.001), whereas no significant effect was observed in PC. Regarding hours worked, shifts of ≥ 7.5 h had a greater effect in hospitals (+37%) compared to PC (+24%). Consistent with our findings, Bae [6] reported that the relationship between work shifts and turnover was significant only in hospitals, where actual hours worked were associated with increased turnover. Thus, the relationship between work shifts and turnover varies depending on the setting [6].

Regarding individual determinants, younger age (< 35 years), male gender, and holding a master’s degree were consistently linked to higher TI. Holding a master’s degree emerged as the second strongest predictor in our model, aligning with European data where higher proportions of master’s‐educated nurses correlate with increased intention to leave [17]. Higher educational attainment has also been associated with increased TI [3, 11, 17]. Being male was linked to higher TI in our study, consistent with prior research [10, 29]. Evidence on age is inconsistent: some studies report younger nurses as more likely to intend to leave [11, 17, 29], aligning with our results, while others, indicate the opposite [28]. In line with age‐related factors, Sumner et al. [15] highlighted generational conflict as a source of workplace tension arising from the generational diversity within the nursing workforce. Specifically, differences in work values, communication styles, and divergent expectations may contribute to interpersonal conflicts and job dissatisfaction if not properly managed. Limited opportunities for professional development have been associated with TI [3, 17]. In our survey, nurses prioritized such opportunities, though this variable was excluded from the model due to low response rates.

### 5.1. The Role of Care Quality and Perceived Value

Perceptions of care quality and patient safety, combined with the sociodemographic block—particularly AC, which carried the greatest weight reflecting Spain’s decentralized health system—explained a proportion of variance comparable to that of the full model. Perceptions of care quality and patient safety emerged as the most influential block in our model. Consistent with previous literature, key risk factors for TI included negative perceptions of care quality [10, 18, 29] and patient safety [10], frequent patient safety incidents (once or more per week) [26], and the omission of nursing care activities. In line with this, Sasso et al. [10] noted that nurses often felt compelled to leave their tasks outside their professional scope, particularly administrative duties. Similar patterns have been observed in other settings, such as intensive care units (ICUs), where nurses have reported feeling a diminished professional role [29].

These findings can be interpreted within the JD‐R framework [16]. Within this model, factors such as adverse working conditions and insufficient staffing operate as job demands and were significantly associated with TI, consistent with evidence on personnel and equipment shortages [11, 17, 29]. In contrast, the availability of adequate work resources operates as a protective factor, buffering the impact of job demands and contributing to more favorable occupational outcomes [11, 18].

When excessive demands compromise care quality and patient safety, they may erode nurses’ internal resources, thereby increasing the intention to leave. In line with this, our model showed overlap between the care quality and working condition blocks, with care quality exerting a stronger influence. These findings underscore that working conditions are associated with nurses’ perceptions and, in turn, are indirectly linked to their intention to leave the profession. Supporting this interpretation, Senek et al. [19] excluded patient load from their final model but suggested that permanent staff shortages and overtime could act as indirect indicators of turnover risk. Despite the JD‐R framework providing a robust theoretical basis for these associations, the cross‐sectional design of our study precludes the establishment of definitive causal relationships between these factors and TI.

In contrast, a 2013 European study found no association between staffing adequacy, nurse–patient ratios, or perceived care quality and safety with nurses’ intention to leave the profession [28]. This discrepancy may reflect changes in working conditions or evolving professional expectations over time.

A previous study highlighted the interactions influencing identifying contributing factors at multiple levels, at the macro‐level, encompassing negative public perceptions and the impact of education on expectations about nursing, at the meso‐level, including limited autonomy, administrative burdens, and poor work‐life balance, and at the micro‐level, such as unmet expectations regarding nursing [15]. A key driver of TI appears to be the perceived imbalance between professional effort and rewards. In our study, nurses expressing intent to leave most frequently cited job insecurity (56.54%) and lack of professional recognition (31.54%) as primary reasons. These results align with a European study of ICU nurses, which identified improved pay and benefits, better staffing, and enhanced recognition as major factors motivating departure [29] as well as with another study conducted in Singapore, where one‐third of nurses reported contemplating leaving the profession, citing reasons such as feeling undervalued, low salary, excessive pressure, and obstacles to career advancement [15].

### 5.2. Implications for Policy and Workforce Planning

Our findings indicate that the primary drivers of nurses’ intention to leave are the interconnected factors of quality of care and working conditions. This underscores the urgent need to enhance working conditions in healthcare institutions, as these modifiable factors are pivotal for policymakers and workforce planners. In the healthcare sector, 77% of staff turnover has been considered avoidable and attributable to factors under the control of the institution. Healthcare systems require adequate resources to address increasing care complexity, ensuring a sufficient supply of highly qualified professionals in supportive environments [10]. Public policies should prioritize the allocation of human, material, and financial resources to recruit and appropriately classify nursing staff based on their training levels and competencies [19].

To mitigate high turnover rates across Europe, tailored strategies must be designed and implemented [29]. This study offers an authoritative basis for shaping national human resources policies and retention strategies. Based on the risk factors identified in our multivariate model and previous literature about retention strategies, we propose the following concrete actions [16].

### 5.3. Short‐Term Actions

• Resource adequacy: Ensure sufficient human and material resources to enable nurses to deliver high‐quality care (OR = 1.71) and maintain patient safety (OR = 1.81), particularly in complex care settings.
• Nurse safe staffing: Implement mandatory nurse‐to‐patient limits, specifically targeting the identified threshold of fewer than 14 patients per nurse (OR = 0.89). Transition toward acuity‐based staffing allocation through a national patient complexity framework that enables dynamic adjustments based on individual care requirements and workload intensity.
• Flexible scheduling: Develop incentive or flexibility programs for evening shifts, which showed significantly higher odds of TI compared to morning shifts (OR = 1.25).

### 5.4. Medium/Long‐Term Actions

• Policy harmonization: Reduce regional disparities in working conditions, addressing the observed effect of AC on TI in this study and ensuring the sustainability of the national healthcare system.
• Targeted retention programs: Design specific mentoring and transition plans for younger cohorts who showed higher intentions to leave (OR = 1.21).
• Professional recognition (career ladder): Establish clearly defined professional categories with appropriate classification based on training level, alongside salary adjustments for higher educational levels, specifically, master’s‐educated nurses (OR = 1.40).
• Job security: Improve transition from temporary or interim contracts to permanent positions, directly addressing the higher risk of TI associated with job instability (OR = 1.33).

While some interventions have been evaluated, evidence of their effectiveness remains inconclusive [19]. Future research should be adapted to the unique characteristics and needs of specific contexts, particularly in European settings [10]. Further studies are needed to develop methodologically rigorous and context‐sensitive strategies based on the identified factors associated with nurses’ TI and to evaluate their effectiveness over time [11, 15]. These strategies should be tested and implemented in real‐world settings to strengthen workforce retention, ensure the sustainability of the nursing profession, and enhance the overall quality of care [11].

### 5.5. Methodological Issues

Interpretation of the results must consider the heterogeneity in definitions, measurements (item/questionnaire/scale), level, and temporal framing of TI across studies [13]. TI can operate at multiple levels: micro (leaving a specific job or unit), meso (leaving the institution or clinical setting), or macro (leaving the profession entirely) [3, 15, 18]. This study focused on macro‐level TI from the nursing profession, limiting direct comparisons with research from countries like the UK, which often examines meso‐level turnover [19, 29]. For additional details, refer to Supporting file 4: Tables 1 and 2.

The development and standardization of the concept of TI, along with the validation of a measurement that can be applied consistently across contexts, including temporal framing, remains insufficiently addressed. Addressing these complexities through context‐aware research will be essential to develop effective, evidence‐based strategies to enhance nurse retention [15]. Future studies should focus on the development of predictive models for actual turnover from the nursing profession. In addition, longitudinal research is needed not only to establish causal relationships between work environment and organizational factors and TI but also to clarify how macro‐level policy reforms translate into daily work experiences.

## 6. Limitations

This study has several limitations. First, its cross‐sectional design precludes causal inferences regarding the associations between the examined factors and TI. In this context, the analysis was exploratory rather than predictive, and the Nagelkerke *R* ^2^ value (0.13) suggests that a considerable proportion of the variance in TI remains unexplained by the variables included in the model. Second, standardized instruments were not used for data collection. Measuring TI using a single dichotomous item may limit sensitivity to different degrees of intention [13, 15]. Despite its simplicity, this measure has been validated in prior research as a robust predictor of actual turnover [10]. Its use is further justified by the challenges involved in accessing large‐scale turnover data [37]. Moreover, the questionnaire was developed through expert consensus and piloted in a homogeneous group of nurses to enhance content validity. Third, self‐report bias is a potential concern, given the self‐administered nature of the data collection, and the length of the questionnaire may have contributed to missing data. Fourth, responses were based on participants’ most recent shift, which may not capture typical workload patterns. Fifth, the categorization of continuous variables may have entailed some loss of information, but this strategy was adopted to facilitate interpretability and comparability across groups.

Sixth, although the very large overall sample size provides substantial statistical power, it also increases the likelihood that even negligible effect sizes achieve statistical significance. For this reason, interpretation prioritized effect sizes (ORs) and their managerial relevance rather than sole reliance on statistical significance [38]. Additionally, the internal distribution of the sample was heterogeneous. Some subgroups represented less than 5% of the sample (e.g., ages 55–60 years, doctoral‐level education, social healthcare settings, relief contracts, 24‐hour shifts, or EIR), resulting in wider CIs and reduced precision for those specific estimates. Conversely, the high representation of nurses under 35 (50%) and those with master’s degree (29%), groups linked to higher turnover odds, may have led to an overestimation of the overall TI rate. This could partially explain why TI in our study is higher than that in other European studies reporting lower rates. This potential overestimation should be considered when interpreting the findings, as the sample distribution may not fully reflect the composition of the nursing workforce in Spain. Finally, backward stepwise selection was used to identify the final model. To mitigate the associated risks of model instability and overfitting, multicollinearity was assessed using VIF, and model performance was evaluated through AIC comparisons and goodness‐of‐fit statistics.

Despite these limitations, this study advances understanding of factors influencing nurses’ TI from the profession in Spain. The findings of this study may provide insights into nurse turnover across different specific care settings. The findings underscore the pressing need for investments in healthier work environments and multifaceted strategies to mitigate nursing workforce attrition.

## 7. Conclusions

Our results reveal a high prevalence of TI in Spain, placing the country among those at highest risk in Europe. These findings provide novel insights into the factors influencing nurses’ TI from the profession across care settings, particularly those related to quality of care and patient safety, highlighting that working conditions shape nurses’ perceptions and indirectly affect their intention to leave. Region, contract type, and shift patterns emerge as directly modifiable policy levers. Addressing these institutional factors is urgently needed to promote healthier, context‐sensitive work environments.

## Author Contributions

Conceptualization: Lucia Rocio Camacho‐Montaño, Sonia Betsabé Gómez‐Arribas, Maria Teresa Moreno‐Casbas, Paloma Calleja‐Toledano, Alda Recas‐Martín, and Javier Padilla‐Bernáldez; methodology: Lucia Rocio Camacho‐Montaño, Sonia Betsabé Gómez‐Arribas, Maria Teresa Moreno‐Casbas, Andrea Ropero‐Sánchez, and Leticia Bernués‐Caudillo; data analysis and interpretation: Lucia Rocio Camacho‐Montaño and Javier Zamora; manuscript writing: Lucia Rocio Camacho‐Montaño, Sonia Betsabé Gómez‐Arribas, and Andrea Ropero‐Sánchez; critical revisions: Maria Teresa Moreno‐Casbas and Javier Padilla‐Bernáldez; supervision: Maria Teresa Moreno‐Casbas and Javier Padilla‐Bernáldez; and final approval: all authors.

## Funding

This research did not receive any specific grant from funding agencies in the public, commercial, or not‐for‐profit sectors.

## Conflicts of Interest

The authors declare no conflicts of interest.

## Supporting Information

Additional supporting information can be found online in the Supporting Information section.

## Supporting information


**Supporting Information 1** STROBE Statement—Checklist of items that should be included in reports of cross‐sectional studies. Contains the STROBE checklist for cross‐sectional studies.

**Supporting Information 2** Supporting file 1: Figure 1. Percentage of participating nurses by autonomous communities. Supporting file 1: Figure 2. Descriptive analysis of nurses’ turnover intention over the next 10 years (broken down by 2, 3, 5, and 10 years). Supporting file 2: Table 1. Detailed post hoc univariate analyses comparing proportions by turnover intention across all categories. Supporting file 3: Table 1. Detailed logistic regression analysis of factors associated with nurses’ turnover intention, including all variables and their categories. Supporting file 3: Figure 1. Logistic regression model for turnover intention, stratified by type of institution. Supporting file 4: Summary of studies on turnover intention. Table 1. Studies conducted in European countries. Supporting file 4: Table 2. Studies conducted in non‐European countries.

## Data Availability

The data supporting the findings of this study are available from the corresponding authors upon reasonable request.
