# Supplementary material for: Turnover Intention and Its Relationship With Work‐Related Factors Among Nurses in Spain: A Cross‐Sectional Study
Source: J Nurs Manag. 2026 May 9;2026:7697811. doi: 10.1155/jonm/7697811 (PMC13157329; doi:10.1155/jonm/7697811)
Supplement: Supplementary file 2 — Supporting Information 2 Supporting file 1: Figure 1. Percentage of participating nurses by autonomous communities. Supporting file 1: Figure 2. Descriptive analysis of nurses’ turnover intention over the next 10 years (broken down by 2, 3, 5, and 10 years). Supporting file 2: Table 1. Detailed post hoc univariate analyses comparing proportions by turnover intention across all categories. Supporting file 3: Table 1. Detailed logistic regression analysis of factors associated with nurses’ turnover intention, including all variables and their categories. Supporting file 3: Figure 1. Logistic regression model for turnover intention, stratified by type of institution. Supporting file 4: Summary of studies on turnover intention. Table 1. Studies conducted in European countries. Supporting file 4: Table 2. Studies conducted in non‐European countries. [file JONM-2026-7697811-s002.docx]

**Supplementary file 1**. Detailed results of the descriptive analysis.

**Figure 1.** Descriptive analysis of study participants by Autonomous Community.
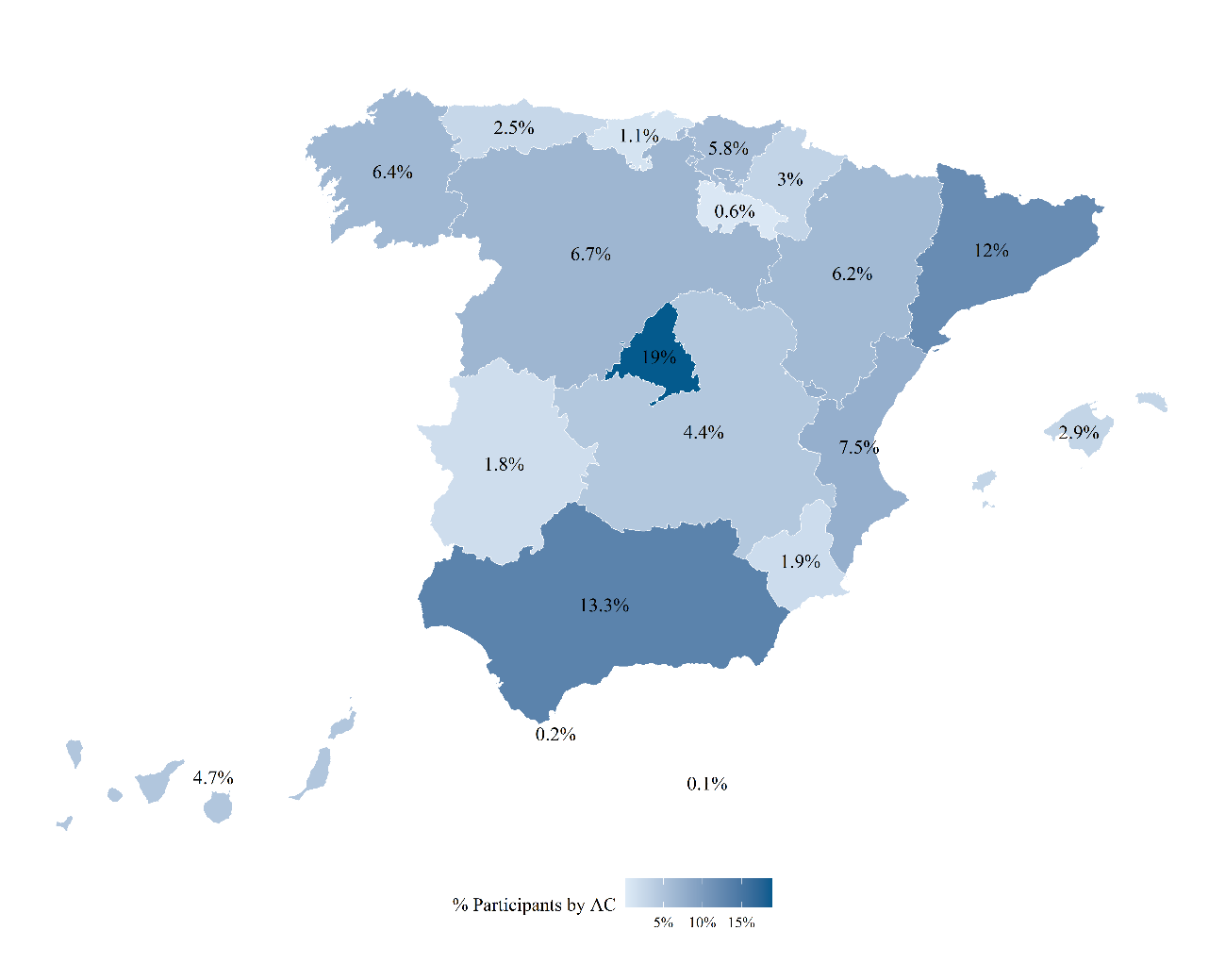


**Figure 2.** Descriptive analysis of nurses’ turnover intention within the next 10 years.


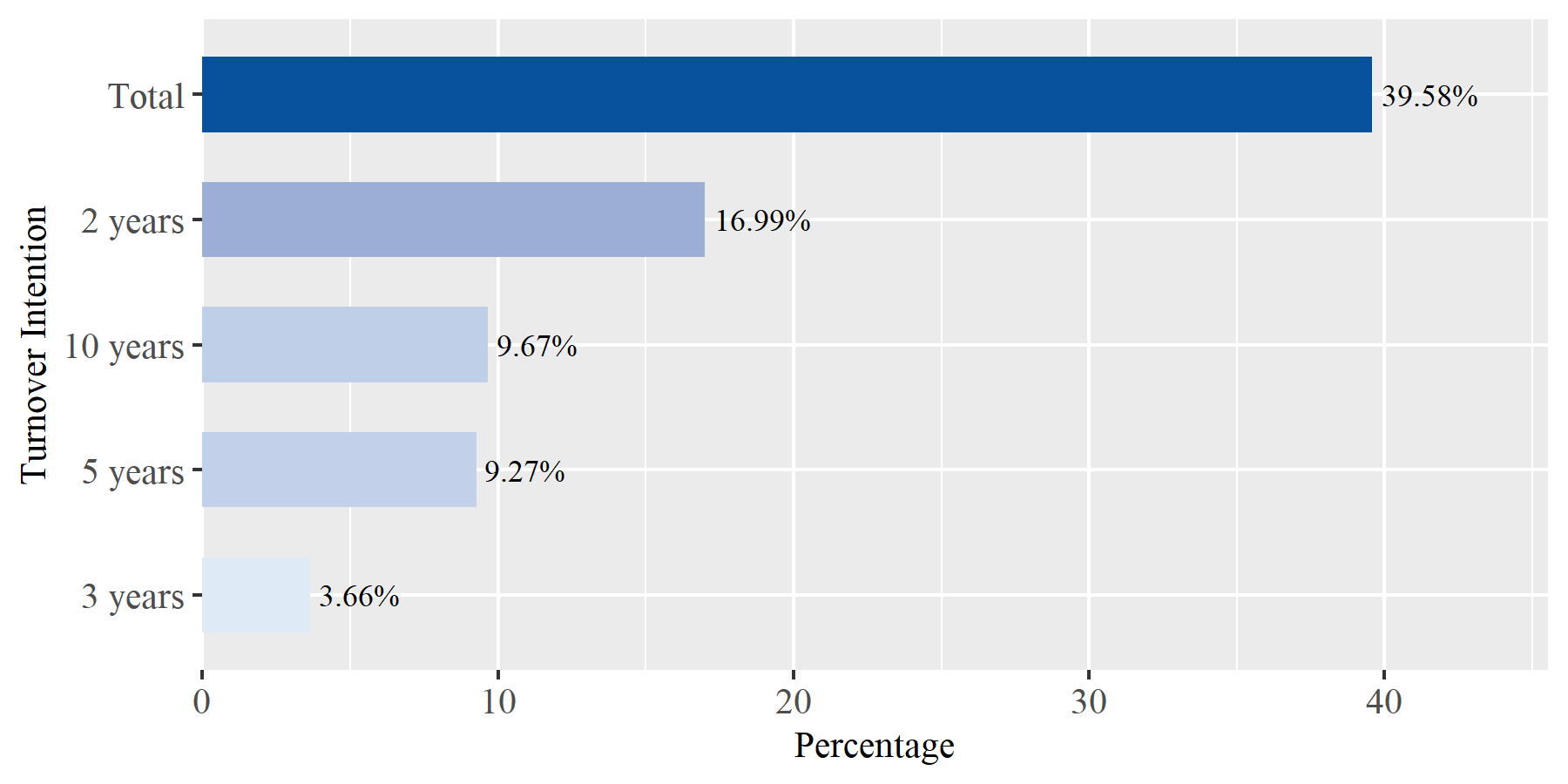


**Supplementary file 2.** Detailed post hoc univariate analyses comparing proportions by turnover intention.

Table 1. Post hoc analysis

| **Variable** | **Comparison** | **Adjusted p-value** | **Significant** | |
| --- | --- | --- | --- | --- |
| Gender | Female - Male | 0.000 | * |  |
| Age | 0-34 - 35-54 | 0.000 | * |  |
|  | 0-34 - 55-60 | 0.000 | * |  |
|  | 35-54 - 55-60 | 0.000 | * |  |
| Care setting | Emergency Services - Hospital | 0.770 |  |  |
|  | Emergency Services - Primary Care | 0.034 | * |  |
|  | Emergency Services - Social-healthcare | 0.039 | * |  |
|  | Hospital - Primary Care | 0.000 | * |  |
|  | Hospital - Social-healthcare | 0.013 | * |  |
|  | Primary Care - Social-healthcare | 0.620 |  |  |
| Current position | Generalist - Specialist | 0.000 | * |  |
| Educational level | Bachelor's degree - Doctorate | 0.742 |  |  |
|  | Bachelor's degree - EIR | 0.001 | * |  |
|  | Bachelor's degree - Master's degree | 0.000 | * |  |
|  | Doctorate - EIR | 0.386 |  |  |
|  | Doctorate - Master's degree | 0.042 | * |  |
|  | EIR - Master's degree | 0.000 | * |  |
| Shift | 12-hour shift - 24-hour shift | 0.011 | * |  |
|  | 12-hour shift - Evening | 0.000 | * |  |
|  | 12-hour shift - Morning | 0.000 | * |  |
|  | 12-hour shift - Night | 1.000 |  |  |
|  | 24-hour shift - Evening | 0.000 | * |  |
|  | 24-hour shift - Morning | 1.000 |  |  |
|  | 24-hour shift - Night | 0.005 | * |  |
|  | Evening - Morning | 0.000 | * |  |
|  | Evening - Night | 0.002 | * |  |
|  | Morning - Night | 0.000 | * |  |
| Contract type | Interim - Permanent | 0.000 | * |  |
|  | Interim - Reinforcement | 0.390 |  |  |
|  | Interim - Temporary | 0.004 | * |  |
|  | Permanent - Reinforcement | 0.000 | * |  |
|  | Permanent - Temporary | 0.000 | * |  |
|  | Reinforcement - Temporary | 0.877 |  |  |
| Area of practice | Both - Rural | 0.000 | * |  |
|  | Both - Urban | 0.855 |  |  |
|  | Rural - Urban | 0.000 | * |  |
| Hours | < 7.5 hours - ≥ 7.5 hours | 0.000 | * |  |
| Ratio: Number of patients | ≥ 21 - 0-7 | 0.000 | * |  |
|  | ≥ 21 - 14-20 | 0.027 | * |  |
|  | ≥ 21 - 8-13 | 0.004 | * |  |
|  | 0-7 - 14-20 | 0.027 | * |  |
|  | 0-7 - 8-13 | 0.096 |  |  |
|  | 14-20 - 8-13 | 0.521 |  |  |
| AC | Andalucía - Aragón | 0.222 |  |  |
|  | Andalucía - Canarias | 0.000 | * |  |
|  | Andalucía - Cantabria | 1.000 |  |  |
|  | Andalucía - Castilla-La Mancha | 1.000 |  |  |
|  | Andalucía - Castilla y León | 1.000 |  |  |
|  | Andalucía - Cataluña | 0.085 |  |  |
|  | Andalucía - Ceuta | 1.000 |  |  |
|  | Andalucía - Comunidad de Madrid | 0.000 | * |  |
|  | Andalucía - Comunidad Foral de Navarra | 0.000 | * |  |
|  | Andalucía - Comunidad Valenciana | 1.000 |  |  |
|  | Andalucía - Extremadura | 0.323 |  |  |
|  | Andalucía - Galicia | 1.000 |  |  |
|  | Andalucía - Islas Baleares | 1.000 |  |  |
|  | Andalucía - La Rioja | 1.000 |  |  |
|  | Andalucía - Melilla | 1.000 |  |  |
|  | Andalucía - País Vasco | 1.000 |  |  |
|  | Andalucía - Principado de Asturias | 1.000 |  |  |
|  | Andalucía - Región de Murcia | 0.001 | * |  |
|  | Aragón - Canarias | 0.000 | * |  |
|  | Aragón - Cantabria | 1.000 |  |  |
|  | Aragón - Castilla-La Mancha | 1.000 |  |  |
|  | Aragón - Castilla y León | 1.000 |  |  |
|  | Aragón - Cataluña | 0.000 | * |  |
|  | Aragón - Ceuta | 1.000 |  |  |
|  | Aragón - Comunidad de Madrid | 0.000 | * |  |
|  | Aragón - Comunidad Foral de Navarra | 0.004 | * |  |
|  | Aragón - Comunidad Valenciana | 1.000 |  |  |
|  | Aragón - Extremadura | 1.000 |  |  |
|  | Aragón - Galicia | 0.010 | * |  |
|  | Aragón - Islas Baleares | 0.517 |  |  |
|  | Aragón - La Rioja | 1.000 |  |  |
|  | Aragón - Melilla | 1.000 |  |  |
|  | Aragón - País Vasco | 1.000 |  |  |
|  | Aragón - Principado de Asturias | 1.000 |  |  |
|  | Aragón - Región de Murcia | 1.000 |  |  |
|  | Canarias - Cantabria | 0.010 | * |  |
|  | Canarias - Castilla-La Mancha | 0.000 | * |  |
|  | Canarias - Castilla y León | 0.000 | * |  |
|  | Canarias - Cataluña | 0.310 |  |  |
|  | Canarias - Ceuta | 1.000 |  |  |
|  | Canarias - Comunidad de Madrid | 1.000 |  |  |
|  | Canarias - Comunidad Foral de Navarra | 0.000 | * |  |
|  | Canarias - Comunidad Valenciana | 0.000 | * |  |
|  | Canarias - Extremadura | 0.000 | * |  |
|  | Canarias - Galicia | 0.024 | * |  |
|  | Canarias - Islas Baleares | 0.109 |  |  |
|  | Canarias - La Rioja | 0.063 |  |  |
|  | Canarias - Melilla | 1.000 |  |  |
|  | Canarias - País Vasco | 0.000 | * |  |
|  | Canarias - Principado de Asturias | 0.000 | * |  |
|  | Canarias - Región de Murcia | 0.000 | * |  |
|  | Cantabria - Castilla-La Mancha | 1.000 |  |  |
|  | Cantabria - Castilla y León | 1.000 |  |  |
|  | Cantabria - Cataluña | 1.000 |  |  |
|  | Cantabria - Ceuta | 1.000 |  |  |
|  | Cantabria - Comunidad de Madrid | 0.004 | * |  |
|  | Cantabria - Comunidad Foral de Navarra | 0.239 |  |  |
|  | Cantabria - Comunidad Valenciana | 1.000 |  |  |
|  | Cantabria - Extremadura | 1.000 |  |  |
|  | Cantabria - Galicia | 1.000 |  |  |
|  | Cantabria - Islas Baleares | 1.000 |  |  |
|  | Cantabria - La Rioja | 1.000 |  |  |
|  | Cantabria - Melilla | 1.000 |  |  |
|  | Cantabria - País Vasco | 1.000 |  |  |
|  | Cantabria - Principado de Asturias | 1.000 |  |  |
|  | Cantabria - Región de Murcia | 1.000 |  |  |
|  | Castilla-La Mancha - Castilla y León | 1.000 |  |  |
|  | Castilla-La Mancha - Cataluña | 0.003 | * |  |
|  | Castilla-La Mancha - Ceuta | 1.000 |  |  |
|  | Castilla-La Mancha - Comunidad de Madrid | 0.000 | * |  |
|  | Castilla-La Mancha - Comunidad Foral de Navarra | 0.001 | * |  |
|  | Castilla-La Mancha - Comunidad Valenciana | 1.000 |  |  |
|  | Castilla-La Mancha - Extremadura | 1.000 |  |  |
|  | Castilla-La Mancha - Galicia | 0.499 |  |  |
|  | Castilla-La Mancha - Islas Baleares | 1.000 |  |  |
|  | Castilla-La Mancha - La Rioja | 1.000 |  |  |
|  | Castilla-La Mancha - Melilla | 1.000 |  |  |
|  | Castilla-La Mancha - País Vasco | 1.000 |  |  |
|  | Castilla-La Mancha - Principado de Asturias | 1.000 |  |  |
|  | Castilla-La Mancha - Región de Murcia | 0.469 |  |  |
|  | Castilla y León - Cataluña | 0.000 | * |  |
|  | Castilla y León - Ceuta | 1.000 |  |  |
|  | Castilla y León - Comunidad de Madrid | 0.000 | * |  |
|  | Castilla y León - Comunidad Foral de Navarra | 0.000 | * |  |
|  | Castilla y León - Comunidad Valenciana | 1.000 |  |  |
|  | Castilla y León - Extremadura | 1.000 |  |  |
|  | Castilla y León - Galicia | 0.101 |  |  |
|  | Castilla y León - Islas Baleares | 1.000 |  |  |
|  | Castilla y León - La Rioja | 1.000 |  |  |
|  | Castilla y León - Melilla | 1.000 |  |  |
|  | Castilla y León - País Vasco | 1.000 |  |  |
|  | Castilla y León - Principado de Asturias | 1.000 |  |  |
|  | Castilla y León - Región de Murcia | 0.428 |  |  |
|  | Cataluña - Ceuta | 1.000 |  |  |
|  | Cataluña - Comunidad de Madrid | 0.005 | * |  |
|  | Cataluña - Comunidad Foral de Navarra | 0.000 | * |  |
|  | Cataluña - Comunidad Valenciana | 0.107 |  |  |
|  | Cataluña - Extremadura | 0.001 | * |  |
|  | Cataluña - Galicia | 1.000 |  |  |
|  | Cataluña - Islas Baleares | 1.000 |  |  |
|  | Cataluña - La Rioja | 1.000 |  |  |
|  | Cataluña - Melilla | 1.000 |  |  |
|  | Cataluña - País Vasco | 0.000 | * |  |
|  | Cataluña - Principado de Asturias | 0.114 |  |  |
|  | Cataluña - Región de Murcia | 0.000 | * |  |
|  | Ceuta - Comunidad de Madrid | 1.000 |  |  |
|  | Ceuta - Comunidad Foral de Navarra | 1.000 |  |  |
|  | Ceuta - Comunidad Valenciana | 1.000 |  |  |
|  | Ceuta - Extremadura | 1.000 |  |  |
|  | Ceuta - Galicia | 1.000 |  |  |
|  | Ceuta - Islas Baleares | 1.000 |  |  |
|  | Ceuta - La Rioja | 1.000 |  |  |
|  | Ceuta - Melilla | 1.000 |  |  |
|  | Ceuta - País Vasco | 1.000 |  |  |
|  | Ceuta - Principado de Asturias | 1.000 |  |  |
|  | Ceuta - Región de Murcia | 1.000 |  |  |
|  | Comunidad de Madrid - Comunidad Foral de Navarra | 0.000 | * |  |
|  | Comunidad de Madrid - Comunidad Valenciana | 0.000 | * |  |
|  | Comunidad de Madrid - Extremadura | 0.000 | * |  |
|  | Comunidad de Madrid - Galicia | 0.000 | * |  |
|  | Comunidad de Madrid - Islas Baleares | 0.023 | * |  |
|  | Comunidad de Madrid - La Rioja | 0.048 | * |  |
|  | Comunidad de Madrid - Melilla | 1.000 |  |  |
|  | Comunidad de Madrid - País Vasco | 0.000 | * |  |
|  | Comunidad de Madrid - Principado de Asturias | 0.000 | * |  |
|  | Comunidad de Madrid - Región de Murcia | 0.000 | * |  |
|  | Comunidad Foral de Navarra - Comunidad Valenciana | 0.000 | * |  |
|  | Comunidad Foral de Navarra - Extremadura | 1.000 |  |  |
|  | Comunidad Foral de Navarra - Galicia | 0.000 | * |  |
|  | Comunidad Foral de Navarra - Islas Baleares | 0.000 | * |  |
|  | Comunidad Foral de Navarra - La Rioja | 1.000 |  |  |
|  | Comunidad Foral de Navarra - Melilla | 1.000 |  |  |
|  | Comunidad Foral de Navarra - País Vasco | 0.001 | * |  |
|  | Comunidad Foral de Navarra - Principado de Asturias | 0.004 | * |  |
|  | Comunidad Foral de Navarra - Región de Murcia | 1.000 |  |  |
|  | Comunidad Valenciana - Extremadura | 0.908 |  |  |
|  | Comunidad Valenciana - Galicia | 1.000 |  |  |
|  | Comunidad Valenciana - Islas Baleares | 1.000 |  |  |
|  | Comunidad Valenciana - La Rioja | 1.000 |  |  |
|  | Comunidad Valenciana - Melilla | 1.000 |  |  |
|  | Comunidad Valenciana - País Vasco | 1.000 |  |  |
|  | Comunidad Valenciana - Principado de Asturias | 1.000 |  |  |
|  | Comunidad Valenciana - Región de Murcia | 0.007 | * |  |
|  | Extremadura - Galicia | 0.034 | * |  |
|  | Extremadura - Islas Baleares | 0.289 |  |  |
|  | Extremadura - La Rioja | 1.000 |  |  |
|  | Extremadura - Melilla | 1.000 |  |  |
|  | Extremadura - País Vasco | 1.000 |  |  |
|  | Extremadura - Principado de Asturias | 1.000 |  |  |
|  | Extremadura - Región de Murcia | 1.000 |  |  |
|  | Galicia - Islas Baleares | 1.000 |  |  |
|  | Galicia - La Rioja | 1.000 |  |  |
|  | Galicia - Melilla | 1.000 |  |  |
|  | Galicia - País Vasco | 0.067 |  |  |
|  | Galicia - Principado de Asturias | 1.000 |  |  |
|  | Galicia - Región de Murcia | 0.000 | * |  |
|  | Islas Baleares - La Rioja | 1.000 |  |  |
|  | Islas Baleares - Melilla | 1.000 |  |  |
|  | Islas Baleares - País Vasco | 1.000 |  |  |
|  | Islas Baleares - Principado de Asturias | 1.000 |  |  |
|  | Islas Baleares - Región de Murcia | 0.003 | * |  |
|  | La Rioja - Melilla | 1.000 |  |  |
|  | La Rioja - País Vasco | 1.000 |  |  |
|  | La Rioja - Principado de Asturias | 1.000 |  |  |
|  | La Rioja - Región de Murcia | 1.000 |  |  |
|  | Melilla - País Vasco | 1.000 |  |  |
|  | Melilla - Principado de Asturias | 1.000 |  |  |
|  | Melilla - Región de Murcia | 1.000 |  |  |
|  | País Vasco - Principado de Asturias | 1.000 |  |  |
|  | País Vasco - Región de Murcia | 0.747 |  |  |
|  | Principado de Asturias - Región de Murcia | 0.751 |  |  |
| Sufficient staffing | No - Yes | 0.000 | * |  |
| Sufficient nursing personnel | No - Yes | 0.000 | * |  |
| Missed nursing care | No - Yes | 0.000 | * |  |
| Quality of care | Fair - Good | 0.000 | * |  |
|  | Fair - Poor | 0.000 | * |  |
|  | Good - Poor | 0.000 | * |  |
| Patient safety | Fair - Good | 0.000 | * |  |
|  | Fair - Poor | 0.000 | * |  |
|  | Good - Poor | 0.000 | * |  |
| Incidents ≥1/week | No - Yes | 0.000 | * |  |

AC: Autonomous Communities, EIR (using the spanish-language acronym): Resident Specialist Nurse.

**Supplementary file 3.** Detailed Logistic Regression Analysis of Factors Associated with Nurses’ Turnover intention

**Table 1.** Full logistic regression model

| **Variable - Level** | **OR** | **Std error** | **z** | **p** | **Lower CI** | **High CI** |
| --- | --- | --- | --- | --- | --- | --- |
| (Intercept) | 0.087 | 0.135 | -18.165 | 0.000 | 0.067 | 0.113 |
| Gender – Male (Ref: Female) | 1.145 | 0.042 | 3.219 | 0.001 | 1.054 | 1.244 |
| Age (Ref:35-54 years) |  |  |  |  |  |  |
| Age 55-60 | 0.962 | 0.082 | -0.475 | 0.635 | 0.817 | 1.129 |
| Age 0-34 | 1.211 | 0.037 | 5.236 | 0.000 | 1.127 | 1.302 |
| Master - Yes (Ref: No) | 1.395 | 0.032 | 10.366 | 0.000 | 1.310 | 1.486 |
| EIR - No (Ref: No) | 1.059 | 0.044 | 1.300 | 0.193 | 0.971 | 1.156 |
| AC (Ref: Navarra) |  |  |  |  |  |  |
| AC - Andalucía | 1.424 | 0.109 | 3.235 | 0.001 | 1.152 | 1.769 |
| AC - Aragón | 1.453 | 0.118 | 3.161 | 0.002 | 1.154 | 1.835 |
| AC - Canarias | 2.237 | 0.122 | 6.576 | 0.000 | 1.763 | 2.850 |
| AC - Cantabria | 1.552 | 0.177 | 2.487 | 0.013 | 1.096 | 2.191 |
| AC - Castilla-La Mancha | 1.436 | 0.125 | 2.906 | 0.004 | 1.127 | 1.836 |
| AC - Castilla y León | 1.466 | 0.117 | 3.269 | 0.001 | 1.168 | 1.847 |
| AC - Cataluña | 1.898 | 0.109 | 5.870 | 0.000 | 1.536 | 2.356 |
| AC - Ceuta | 1.151 | 0.406 | 0.346 | 0.729 | 0.500 | 2.493 |
| AC - Comunidad de Madrid | 2.429 | 0.106 | 8.362 | 0.000 | 1.978 | 2.999 |
| AC - Comunidad Valenciana | 1.513 | 0.116 | 3.558 | 0.000 | 1.207 | 1.905 |
| AC - Extremadura | 1.195 | 0.156 | 1.140 | 0.254 | 0.879 | 1.623 |
| AC - Galicia | 2.008 | 0.117 | 5.949 | 0.000 | 1.599 | 2.532 |
| AC - Islas Baleares | 1.990 | 0.134 | 5.133 | 0.000 | 1.532 | 2.591 |
| AC - La Rioja | 1.297 | 0.229 | 1.136 | 0.256 | 0.822 | 2.019 |
| AC - Melilla | 1.837 | 0.414 | 1.468 | 0.142 | 0.799 | 4.102 |
| AC - País Vasco | 1.362 | 0.119 | 2.599 | 0.009 | 1.081 | 1.723 |
| AC - Principado de Asturias | 1.522 | 0.140 | 3.008 | 0.003 | 1.158 | 2.003 |
| AC - Región de Murcia | 0.893 | 0.157 | -0.718 | 0.473 | 0.656 | 1.214 |
| Care setting (Ref: Hospital) |  |  |  |  |  |  |
| Care setting - Social-healthcare | 0.779 | 0.096 | -2.604 | 0.009 | 0.645 | 0.939 |
| Care setting - Emergency Services | 0.853 | 0.061 | -2.624 | 0.009 | 0.757 | 0.960 |
| Care setting - Primary Care | 1.046 | 0.050 | 0.906 | 0.365 | 0.948 | 1.155 |
| Current position - Generalist (Ref: Specialist) | 1.117 | 0.052 | 2.125 | 0.034 | 1.009 | 1.238 |
| Area of practice - Both (Ref: Rural) | 1.147 | 0.077 | 1.793 | 0.073 | 0.988 | 1.333 |
| Area of practice - Urban | 1.108 | 0.068 | 1.519 | 0.129 | 0.971 | 1.265 |
| Contract (Ref: Permanent) |  |  |  |  |  |  |
| Contract - Interim | 1.235 | 0.042 | 5.032 | 0.000 | 1.138 | 1.342 |
| Contract - Reinforcement | 1.169 | 0.112 | 1.396 | 0.163 | 0.938 | 1.455 |
| Contract - Temporary | 1.333 | 0.045 | 6.449 | 0.000 | 1.221 | 1.455 |
| Shift (Ref: Morning) |  |  |  |  |  |  |
| Shift - 12-hour | 0.907 | 0.056 | -1.740 | 0.082 | 0.812 | 1.012 |
| Shift - 24-hour | 1.004 | 0.093 | 0.045 | 0.964 | 0.836 | 1.205 |
| Shift - Evening | 1.253 | 0.044 | 5.177 | 0.000 | 1.151 | 1.365 |
| Shift - Night | 0.966 | 0.056 | -0.624 | 0.533 | 0.866 | 1.077 |
| Hours - ≥ 7.5 hours (Ref: <7.5) | 1.321 | 0.044 | 6.323 | 0.000 | 1.212 | 1.440 |
| Number of patients (Ref: 0-7) |  |  |  |  |  |  |
| Ratio - ≥ 21 patients | 1.036 | 0.050 | 0.704 | 0.481 | 0.939 | 1.142 |
| Ratio - 14-20 patients | 0.952 | 0.046 | -1.051 | 0.293 | 0.869 | 1.043 |
| Ratio - 8-13 patients | 0.884 | 0.044 | -2.826 | 0.005 | 0.811 | 0.963 |
| Sufficient staffing – No (Ref: Yes) | 1.193 | 0.036 | 4.886 | 0.000 | 1.111 | 1.281 |
| Missed nursing care - Yes (Ref: No) | 1.331 | 0.036 | 7.914 | 0.000 | 1.240 | 1.429 |
| Quality of care (Ref: Good) |  |  |  |  |  |  |
| Quality of care - Fair | 1.441 | 0.042 | 8.781 | 0.000 | 1.328 | 1.563 |
| Quality of care - Poor | 1.706 | 0.099 | 5.383 | 0.000 | 1.405 | 2.073 |
| Patient safety (Ref: Good) |  |  |  |  |  |  |
| Patient safety - Fair | 1.284 | 0.041 | 6.130 | 0.000 | 1.186 | 1.391 |
| Patient safety - Poor | 1.809 | 0.089 | 6.656 | 0.000 | 1.520 | 2.156 |
| Incidents ≥1/week - Yes (Ref: No) | 1.337 | 0.035 | 8.318 | 0.000 | 1.249 | 1.432 |
| Note: Odds Ratios (OR), standard errors (Std error), z-values (z), p-values (p) and confidence intervals (CI) are reported from the logistic regression model assessing factors associated with nurses’ turnover intention from the profession. Statistically significant associations are considered at p < 0.05. Reference categories for categorical variables were female gender, age 35-54 years, Master’s degree “No”, EIR “Yes”, institution “Hospital,” permanent contract, morning shift, <7.5 hours, patient load of 0–7 patients, good ratings for quality of care and patient safety. Variables with OR > 1 indicate increased odds of turnover intention, whereas OR < 1 indicate decreased odds and an OR = 1 indicates no effect. AC: Autonomous Community. EIR (using the spanish-language acronym): Resident Specialist Nurse. | | | | | | |


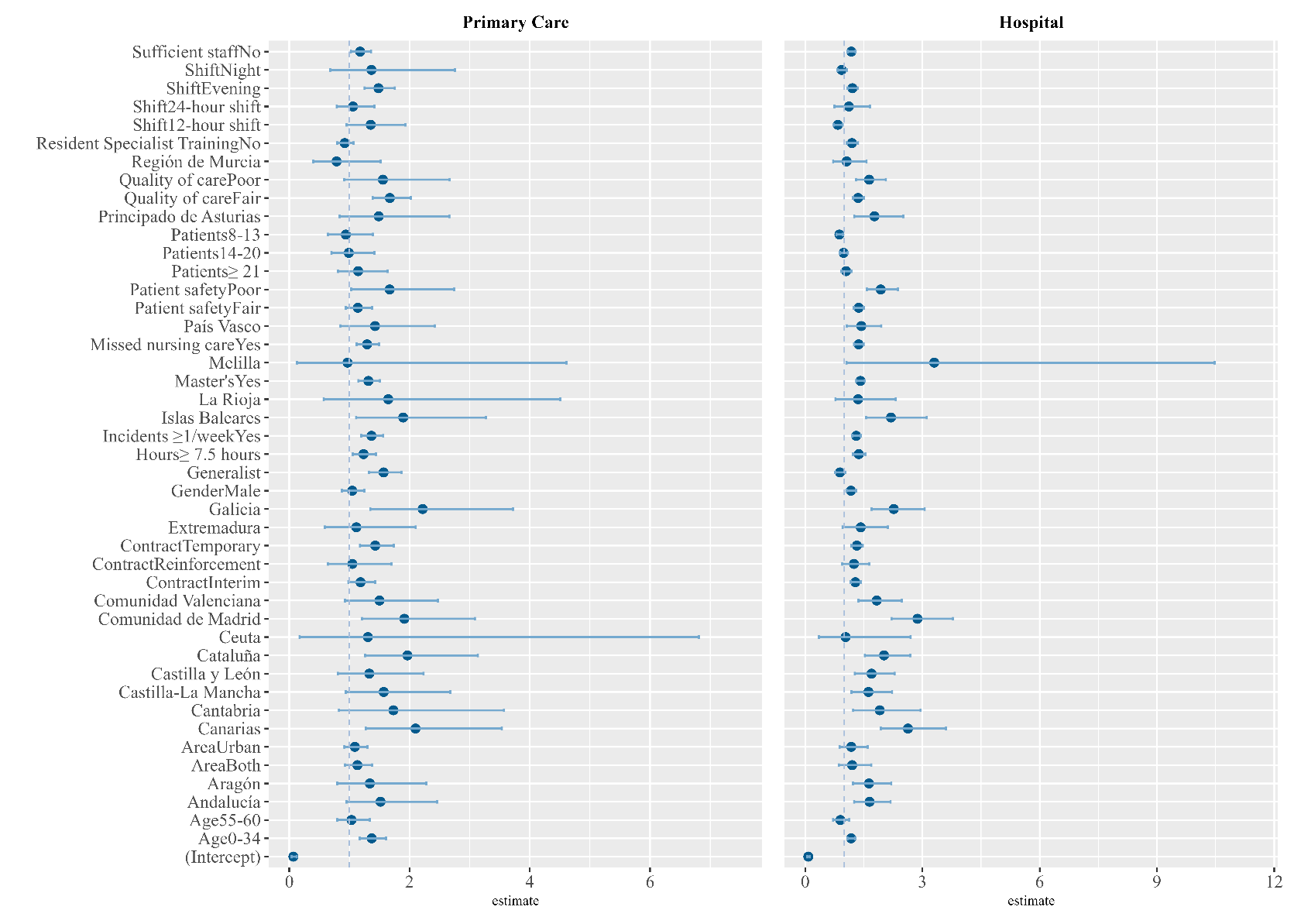
**Figure 1.** Logistic Regression Model for turnover intention, Stratified by type of institution.

**Supplementary file 4.** Summary of studies on turnover intention.

**Table 1.** Studies conducted in European countries.

| Reference | Country | Data collection date | Setting | Sample size | Turnover intention | | |
| --- | --- | --- | --- | --- | --- | --- | --- |
|  |  |  |  |  | **Item/question** | **Level** | **Time frame** |
| Estryn-Béhar et al., 2007 | Belgium, Finland, France, Germany, Italy, Netherlands, Norway, Poland, Slovakia, and the United Kingdom | October 2002 and June 2003 | hospitals | 28561 nurses | 1 item: “How often during the course of the past year have you thought about giving up nursing?” The scale anchors are: “never,” “sometime in a year,” “sometime in a month,” “sometime in a week,” and “every day.” We have interpreted “sometime in a month” or more often as an indicator of frequently considering intent to leave | 15.6% - giving up nursing | the past year |
| Heinen et al., 2013 | Belgium, Finland, Germany, Ireland, the Netherlands, Norway, Poland, Spain, Switzerland and the UK | January 2009-June 2010  (Sermeus et al., 2011) | hospitals | 23159 nurses | if they intended to leave their job due to job dissatisfaction. If they answered in the affirmative, they were asked to differentiate between leaving the hospital/profession. | 33% hospital  9% profession | the next year |
| Ivziku et al., 2025 | Italy | August 2022 and December 2023 | public or private healthcare institutions | 1745 nurses | Nurses’ intentions to leave their current unit, healthcare institution, or the nursing profession | 26.5% unit  22.2% institution  17.7% profession | intend to leave within the next six months |
| Llaurado-Serra et al., 2025 | Cyprus, Poland, Croatia, Romania and Spain | January 2021 and April 2022 | in critical care units | 1033 nurses | Not specified | 21.4 %  11.4 % | the next 3 years  the next 12 months |
| Maniscalco et al., 2024  +  Enea et al., 2024 | Belgium, the Netherlands, Italy, Poland | May 16 and September 30, 2022 | hospitals | 1351 nurses | “I intend to leave my healthcare profession for another job” | 8.9% hospital  13.6% profession | “in the near future” |
| Sasso et al., 2019 | Italy | September to December 2015 | hospitals | 3667 nurses | chances of leaving their hospital job because of job dissatisfaction; nurses who answered “yes” were asked if they would change with another hospital or sector, or leave their nursing profession. | 35.5% hospital  11.7% profession | the next year |
| Senek et al., 2023 | UK  (England, Scotland, Northern Ireland and Wales) | February 8^th^- April 26^th^ 2021 | community nurses working | 533 nurses | intention to leave the job question (yes/no/maybe) | Intend to Leave the current Job:  27.6% yes  35.6% maybe | Not specified |

**Table 2.** Studies conducted in non-European countries.

| Reference | Country | Data collection date | Setting | Sample size | Turnover intention | | |
| --- | --- | --- | --- | --- | --- | --- | --- |
|  |  |  |  |  | **Item/question** | **Level** | **Time frame** |
| Cao et al., 2021 | China | January to March 2018 | hospitals | 12291 | Turnover Intention Scale (Yang et al., 2017): possibility of quitting the present job, motivation to find other employment and possibility of access to external work.  4-point Likert scale: frequently (4), occasionally (3), seldom (2) and never (1). | hospital (institution)  13.97 ± 3.63  (mean ± sd)  (score > 3) to leave their jobs: 9.82% strong intention | Not specified |
| Huang et al., 2024 | China | October to November 2017 | hospitals | 2504 | Turnover intention score;  wo levels according to the median score: low (≤ 15) and high (> 15). | their occupation;  43% - willingness to leave | Not specified |
| Summer et al, 2025 | Singapore | March to June 2023 |  | 479 | I am thinking about changing organization but will continue nursing.  I am thinking about leaving the nursing profession.  Likert scale: from Strongly Agree to Strongly Disagree. | 53% for the organization and 36% for the nursing profession. | Not specified |
| Wang et al., 2022 | China | October to December 2017 | psychiatric hospitals | 2355 | turnover intention questionnaire (TIQ). Each item ranged from 1 (never) to 4 (frequently). The total score was divided into four levels: lower (≤6), low (6–12), high (13–18) and higher (>18) | current job | Not specified |
